# Suppression of Interferon-α Treatment Response by Host Negative Factors in Hepatitis B Virus Infection

**DOI:** 10.3389/fmed.2021.784172

**Published:** 2021-11-24

**Authors:** Jiayi Wang, Lingyao Du, Hong Tang

**Affiliations:** ^1^Center of Infectious Diseases, West China Hospital of Sichuan University, Chengdu, China; ^2^Division of Infectious Diseases, State Key Laboratory of Biotherapy and Center of Infectious Diseases, West China Hospital, Sichuan University, Chengdu, China

**Keywords:** chronic hepatitis B, interferon-α, negative regulators, host factors, non-response

## Abstract

Chronic hepatitis B virus (CHB) infection remains a major global public health issue for which there is still lacking effective curative treatment. Interferon-α (IFN-α) and its pegylated form have been approved as an anti-HBV drug with the advantage of antiviral activity and host immunity against HBV infection enhancement, however, IFN-α treatment failure in CHB patients is a challenging obstacle with 70% of CHB patients respond poorly to exogenous IFN-α treatment. The IFN-α treatment response is negatively regulated by both viral and host factors, and the role of viral factors has been extensively illustrated, while much less attention has been paid to host negative factors. Here, we summarized evidence of host negative regulators and parameters involved in IFN-α therapy failure, review the mechanisms responsible for these effects, and discuss the possible improvement of IFN-based therapy and the rationale of combining the inhibitors of negative regulators in achieving an HBV cure.

## Introduction

Chronic hepatitis B virus (CHB) infection affects more than 290 million people worldwide, with 1.5 million new infections each year, estimated by World Health Organization (1). The lifetime risk of developing hepatocellular carcinoma (HCC) and liver cirrhosis among HBV carriers ranges from 10 to 25% (2) and from 15 to 40% (3), respectively. HBV is a partially double-stranded circular DNA virus belonging to the Hepadnaviridae family (4), first discovered in an Australian aborigine (5). HBV has 10 genotypes (A-J) with nearly 40 sub-genotypes distributed in distinct areas, while the infectious in East Asia are most commonly HBV genotype B and C (3, 6). Current HBV treatment strategies can be categorized into 2 groups: interferons (IFN) and nucleos (t)ide analogs (NAs).

IFNs are a group of signaling proteins released by host cells in response to various pathogens, including viruses, bacteria, and parasites. IFN-α belongs to type I IFNs and has been approved as an anti-HBV therapy. Although with multiple adverse effects and inconvenient administration, IFN-α has the strength of a relatively short interval of treatment, without risk of drug resistance, higher rate of hepatitis B e antigen (HBeAg) seroconversion, and particularly hepatitis B surface antigen (HBsAg) seroclearance, which is unable to reach by current NAs administration (7, 8). Therefore, it is important to identify CHB patients who will benefit from treatment before the start of IFN-α-based therapy. Besides, IFN's efficacy is far less than satisfactory, with only one-third of HBeAg positive CHB patients achieved HBeAg seroconversion after the IFN-α therapy, and even less efficacy was observed in HBeAg negative patients (9). The molecular mechanisms responsible for the failure of IFN-α treatment are not well-understood, but evidence shows that both viral and host factors are involved. Viral factors include HBV genotype, mutations within the HBV genome, and baseline level of viral load. Individuals infected with HBV-C and D (comparing to HBV-A and B) (6, 10), HBV has mutations within the HBV pre-core and/or basal core promoter (PC and/or BCP) region (6, 11–13), and high baseline viral load are tend to be more resistance to IFN-α therapy. The molecular mechanisms underlying the virally mediated resistance to IFN-α have been summarized in another review (14). In addition to the viral factors, host factors play an equally important role in modulating the effectiveness of IFN-α therapy for CHB patients' treatment. Some host molecules function as negative regulators of IFN therapy by inhibiting IFN production or signaling pathways, while several host parameters provide essential information of liver function and are able to predict the efficacy of IFN-α therapy. Therefore, a thorough understanding of the mechanism responsible for host factor-mediated inhibition of IFN therapy is needed for providing therapeutic targets to improve the efficacy of IFN-α treatment in terms of HBV infection. In this review, we summarize host negative regulators that impair IFN-α therapy of HBV infection and host parameters that can predict the IFN-α therapy efficacy, review the underlying mechanisms, as well as discuss the potential therapeutic approaches for controlling HBV infection.

## Classical IFN and ISGs Production Pathways in Response to HBV Infection and Antiviral Activity of IFN-α

Interferon (IFN) was originally discovered in 1957 by Isaacs and Lindenmann, and was named for their ability to interfere with viral replication (15, 16). Three interferon families are discovered—type I, II, and III. The type I IFN family encodes 13 partially homologous IFN-α subtypes in humans, IFN-β and several single gene products (IFN-ε, IFN-τ, IFN-κ, IFN-ω, IFN-δ, and IFN-ζ). The type IFN II family comprises a single gene product, IFN-γ, while the type III IFN family consists of IFN-λ1, IFN-λ2, IFN-λ3 (also known as IL-29, IL-28A and IL-28B, respectively), and IFN- λ4 (17, 18). Type I IFNs are secreted by almost all virus-infected cells including hepatocytes and by specialized blood lymphocytes, while the production of IFN-γ is restricted to immune cells, including natural killer (NK) cells, macrophages, and T cells. IFN-α and pegylated IFN-α have been approved for the treatment of chronic hepatitis B. IFN- α binds to its receptor (IFN-α/β-receptor, IFNAR) leading to the downstream signaling pathway and result in the expression of various IFN-stimulated genes (ISGs), which have multiple functions including anti-viral, anti-proliferation, anti-tumor, and immunomodulation. Some of these directly inhibit virus transcription and translation, others function as host immune modulators by NK cells activation, Dendritic cells (DCs) maturation, CD8+ T-cell augmentation, and B cell response (19). The antiviral effect against HBV infection of IFN was first known in 1976 by giving human fibroblast IFN to patients with HBsAg-positive chronic aggressive hepatitis (20).

The IFN response is initially induced by the recognition of HBV components. Multiple forms of nucleic acid are generated during the HBV life cycle, including double-strand relaxed circular DNA (rcDNA) and covalently closed circular DNA (cccDNA), single-strand RNAs, as well as double-strand RNAs, all of which could stimulate pattern recognition receptors (PRRs) on virus infection. Studies suggested that in the early phase of infection, HBV could activate some PRRs, which in turn stimulate the innate immune response to limit viral replication and clearance (21). For instance, Viral DNA is sensed by DEAD-box protein 41 (DDX41), cyclic GMP-AMP synthase (cGAS), and γ-IFN-inducible protein 16 (IFIT16), leading to the activation of stimulator of IFN genes (STING). RNA is recognized by either Toll-like receptor 3 (TLR3) or cytoplasmic sensors such as retinoic acid-inducible gene-I (RIG-I) and melanoma differentiation-associated gene 5 (MDA5), leading to its association with the mitochondrial antiviral signaling protein (MAVS). Both MAVS recruitment and STING activation lead to TANK-binding kinase 1 (TBK1) phosphorylation, which can activate IFN regulatory factor 3 (IRF3) and IRF7, two important transcriptional factors required for induction of type I IFN (22). In the HBV setting, Kupffer cells and liver parenchymal cells can recognize HBV components through intracellular PRRs such as TLRs and RIG-1-like receptors (RLRs), which induce the production of type I IFN (23). Several studies have also suggested TLR, RIG-I, STING, and myeloid differentiation primary response 88 (MyD88)-dependent pathway may participate in HBV-mediated IFN-induction in human cells (24, 25) and the pathways are meanwhile blocked by HBV polymerase in infected cells (26–28). In the MYD88-dependent pathway, MYD88 recruits a set of signal cascades such as MAPK and NF-κB through receptor-interacting serine/threonine protein kinase (RIPK/RIP) (29). After IFN-α was secreted by infected cells and blood cells, it will recruit in a pathway to induce ISGs expression.

The IFN-α signaling cascade is initiated through interactions with a multisubunit cell surface receptor consisting of two distinct receptor subunits, IFN-α receptor 1 (IFNAR1) and IFNAR2, leading to the activation of IFNAR-associated tyrosine kinases, Janus kinases 1 (JAK1) and tyrosine kinase 2 (Tyk2), which phosphorylate both IFNAR1 and IFNAR2 subunits, followed by the activation and phosphorylation of signal transducer and activator of transcription factors (STATs) in the canonical IFN signaling. STAT1 and STAT2 then form heterodimers and are joined by an Interferon regulatory factor 9 (IRF9) to form an active transcription factor complex known as IFN-stimulated gene factor 3 (ISGF3). ISGF3 translocates into the nucleus and binds to the IFN-stimulated response element (ISRE) to initiate transcription of ISGs to affect HBV replication or modulate host immune response (30). IFN-α exerts antiviral activity against HBV by both inducing antiviral gene products that inhibit viral replication in hepatocytes and by modulating the host immune system. Many ISGs are known to inhibit HBV replication at different steps, including inhibiting cccDNA transcription and HBV nucleocapsid formation, suppressing the activity of HBV enhancers, as well as control the HBV replication at the post-transcriptional level (31).

The other complex formed by STAT1 homodimers binds to the GAS motif and mainly active pro-inflammatory gene expression. After STAT1 homodimers bind to the GAS enhancer elements in the promoters of IFN-stimulate genes, genes encoding pro-inflammatory cytokines and apoptotic factors are induced (32). Type I IFN can also activate STAT3 homodimers and result in the production of both pro-inflammatory cytokines and anti-inflammatory cytokines (such as interleukin-10 (IL-10). In the non-canonical IFN signaling, IFN can induce a set of genes independent of STATs, including MAPKs and PI3K, to initiate ISGs transcription (33). In a STAT-independent manner, type I IFNs activate both p38, which is an upstream activator of several genes regulated by ISREs and GAS elements, and mammalian target of rapamycin (mTOR), which regulates mRNA translation (34).

Type I IFN can activate other important members of innate immunity, including natural killer (NK) cells and natural killer T (NKT) cells, recruiting them to the infected tissues and recognize infected hepatocytes. These immune cells secrete cytokines that promote intracellular HBV clearance and induce apoptosis. Pegylated IFN-α treatment enhanced recovery of memory T cells in CHB patients by down-regulating inhibitory receptors and up-regulating effector molecules (35). The antiviral effect against HBV of IFN and its immunomodulatory function have been finely summarized in several reviews (22, 31, 34).

## Negative Regulators in IFN Induction and Signaling Pathway

Viruses could disrupt IFN responses by co-opting negative regulatory systems or use the antiviral system to their advantage (36), for example, HBV could hijack several ISGs either to facilitate its replication or impede innate immune response to HBV. Although viral component recognition by PRRs is essential for effective antiviral immune responses, the inflammatory immune response, including type I IFN production, has to be tightly regulated to prevent advert immune-related pathologies. Because activation of those inhibitory processes can cause IFN-α therapy suppression, understanding the molecular basis and identify related host negative regulators could provide targets for improving IFN-α treatment efficacy.

**Figure 1:**
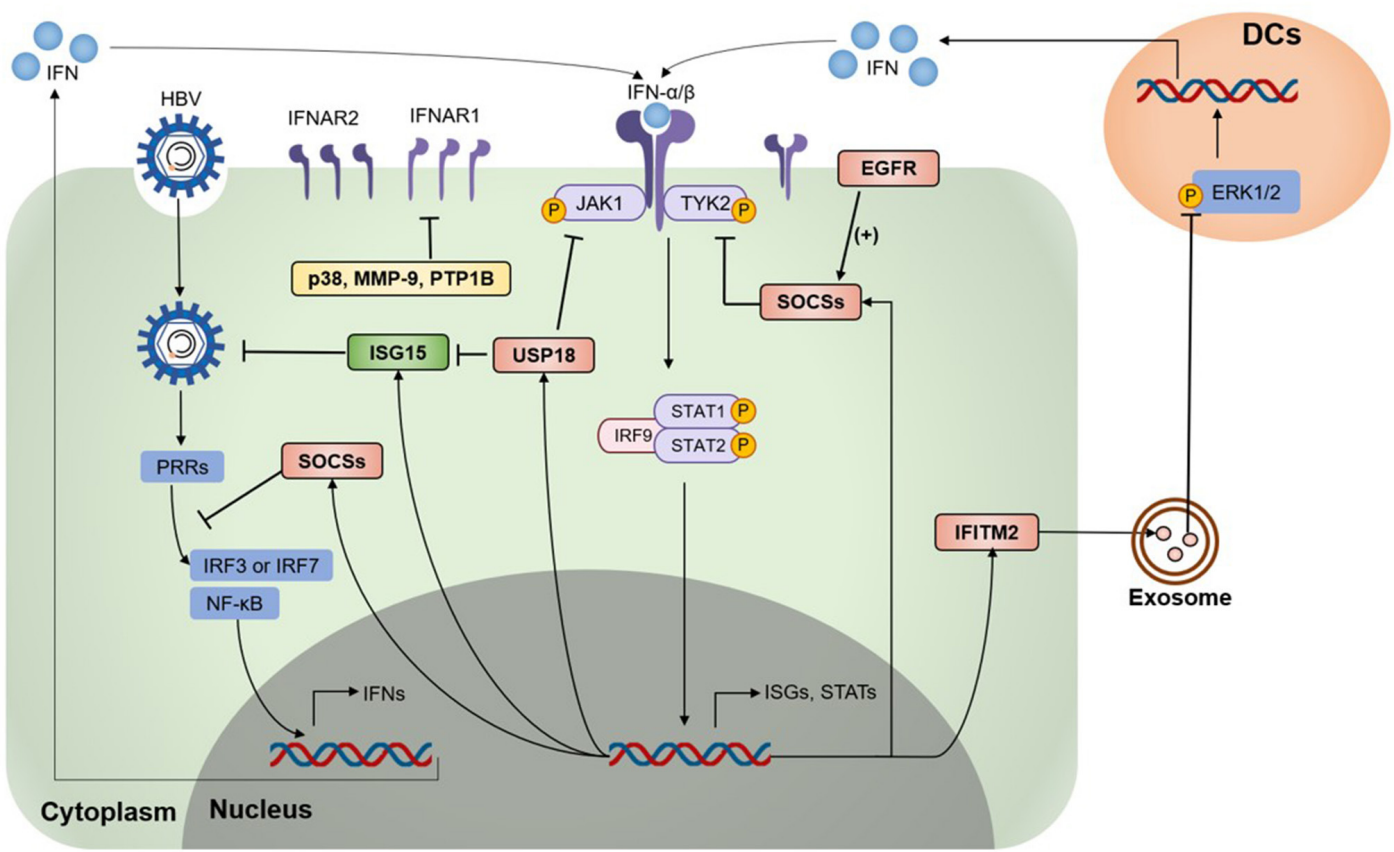
Type I IFN signaling is down-regulated by negative regulators. Various negative regulators cross-regulate the type I interferon (IFN) response, which modulates the expression levels and activation states of IFN signaling components. On HBV infection and invasion, pattern-recognition receptors (PRRs) recognize HBV and activate the downstream pathway to induce IFN expression. IFNAR1/2 recognize type I IFN and activate Janus kinases 1 (JAK1) and tyrosine kinase 2 (Tyk2), followed by the activation and phosphorylation of signal transducer and activator of transcription factors (STATs), leading to the expression of various IFN-stimulated genes (ISGs). Several ISGs were identified as negative regulators of the IFN signaling pathway, including the suppressor of cytokine signaling (SOCS) proteins, ubiquitin-specific protease 18 (USP18), Interferon-induced transmembrane protein 2 (IFITM2), p38 mitogen-activated kinases (p38 MAPKs), matrix metalloproteinase 9 (MMP-9), epidermal growth factor receptor (EGFR), protein tyrosine phosphatases 1 B (PTP1B), and some regulators awaiting exact mechanisms. HBV, hepatitis B virus; IRF3, IFN regulatory factor 3; IRF7, IFN regulatory factor 7; ERK1/2, extracellular signal-regulated kinase 1/2; DCs, dendritic cells.

Studies showed that IFNAR1 and IFNAR2 in peripheral blood mononuclear cells and lymphocytes increased in CHB patients, but their level had a positive correlation with HBV-DNA in liver tissue (37). Several negative regulators mainly functioning to suppress IFNs expression or disturb the function or expression of IFNAR, thereby inhibit the JAK-STAT pathway and ISGs production (Figure 1). The related negative regulators are summarized in Table 1.

**Table 1:** Involvement and the possible underlying mechanisms of host negative regulators in the inhibition of IFN-α therapy.

**Host negative regulators**	**Possible underlying mechanisms**
**Negative regulators in IFN signaling**	
p38 MAPKs	Attenuate IFN-α through down-regulating IFNAR1 by UPR-stimulated priming phosphorylation of IFNAR1 (38).
MMP-9	Inhibit IFN-α signaling *via* the promotion of IFNAR1 degradation through lysosome pathways (39).
IFITMs	IFITM2 inhibits the antiviral activity through inhibiting IFN-α synthesis by inhibiting phosphorylation of ERK, TBK1, and IRF3 (40).
USP18	USP18 attenuates IFN-α signaling *via* both ISGylating ISG15 (41) and compete with JAK1 for binding to the IFNAR2 (42).
SOCSs (SOCS 1-3 and CIS)	SOCS 1-3 and CIS suppress the efficacy of IFN-α therapy by suppress IFN-α production (43, 44) and inhibiting JAK-STAT pathway to reduce the duration of antiviral genes expression (45, 46).
EGFR	EGFR affects IFN-α therapy *via* the induction of SOCS3 (47).
SHP2	SHP2 inhibits IFN-α signaling by a PKCβ-dependent pathway (48, 49).
PTP1B	PTP1B may inhibit IFN-α signaling *via* binding and dephosphorylating IFNAR1 (50).
NT5C3	NT5C3 may inhibit the IFN-α therapeutic effect by binding and sequestering miR-122, which is an anti-HBV molecule (51).
**Cytokines, chemokines and non-coding RNAs**	
Peripheral IFNAR	The mechanisms are not clear and may related to oxidative stress and the disturbance of IFNAR function (37, 52).
TNF-α	TNF-α may inhibit IFN-α therapy efficacy *via* the induction of SOCS3 and SHP2 (53).
IL-10	IL-10 inhibits IFN-α signaling *via* the induction of SOCS (54); IL-10 suppresses IFN-α and IFN-γ production (55, 56).
IL-8	IL-8 suppresses the antiviral efficacy and signaling of IFN-α (57).
IL-28B genetic polymorphisms	IL-28B rs12979860 CC genotype and rs8099917 TT genotype indicate better treatment response, mechanisms are undiscovered (58).
miR-146a	miR-146a inhibits IFN-α *via* suppression of STAT1 and attenuation of ISGs production (59, 60).
miR-3613-3p	miR-3613-3p decreases the expressions of IFN-α and IFN-β through targeting CMPK1 (61).
miR-21	miR-21 promotes an anti-inflammatory response by increasing IL-10 (61).
circRNA hsa-circ-0004812	circRNA hsa-circ-0004812 impairs IFN-induced immune response by regulating FSTL1 (62).

*MAPKs, mitogen-activated kinases; IFNAR, interferon-α/β-receptor; UPR, unfolded protein response; MMP-9, Matrix metalloproteinase 9; IFITMs, Interferon-induced transmembrane proteins; ERK, extracellular signal-regulated kinase; TBK1, TANK-binding kinase 1; IRF3, interferon regulatory gactor 3; USP18, ubiquitin-specific protease 18; SOCS, suppressor of cytokine signaling; CIS, cytokine-inducible SH2; EGFR, epidermal growth factor receptor; PKCβ, protein kinase Cβ; PTPs, Protein tyrosine phosphatases; STAT1, signal transducer and activator of transcription factor 1; ISGs, IFN-stimulated genes; CMPK1, cytidine monophosphate kinase 1; FSTL1, Follistatin-related protein 1*.

### p38 MAPKs

Mammalian p38 mitogen-activated kinases (MAPKs) are activated by cellular stresses and inflammatory cytokines and are critical for normal immune and inflammatory response (63). Several studies showed HBV infection could activate mitogen-activated protein kinases (MAPKs), including p38 MAPK kinases, c-Jun N-terminal kinase (JNK), and extracellular signal-regulated kinases (ERKs) (64–67). p38 MAPK, but not JNK or ERK1/2, was significantly phosphorylated in Huh7 cells after HBV infection and thereby promote intracellular HBV replication (68) with the involvement of STAT3 (67). p38 MAKP also acts as a negative regulator of type I IFN pathway through unfolded protein response (UPR)-stimulated priming phosphorylation of IFNAR1, ensuring IFNAR1 down-regulation and type I IFN signaling attenuation (38). Given this knowledge as well as the important role of HBV in p38 MAPKs production, we hypothesized that negative regulation of p38 MAPKs should stabilize IFNAR1 and improve IFN therapy efficacy against chronic HBV infection, which requires further investigation.

### MMP-9

Matrix metalloproteinase 9 (MMP-9) is expressed in normal leukocyte and transformed cells, and is associated with IFNAR1 degradation (39). IFNAR1 degradation can be promoted by either ligand binding and cellular signaling induced ubiquitination-dependent pathway, or Ser532 phosphorylation and p38 kinase activity requiring ligand-independent way (69). MMP-9 can be activated by HBV in CHB patients (70, 71) and in turn facilitates HBV replication through attenuating IFN/JAK/STAT signaling, interacting with IFNAR1 to promoting its phosphorylation, ubiquitination, subcellular distribution, and degradation of IFNAR1 through lysosome pathways in Ser532 phosphorylation and p38 independent manner, as well as blocking its binding to IFN-α (39). However, a recent study found type I IFN strongly promotes the clearance of MMPs (72), thus, further study is needed to investigate the role of MMPs in the IFN-α therapy mediated antiviral efficacy against HBV infection.

### IFITMs

Interferon-induced transmembrane proteins (IFITMs) are a family of small proteins that localize in the plasma and endolysosomal membranes (73) and were originally described based on their expression after IFN therapy (74). The human IFITM family comprises five members, including immune-related IFITM1, IFITM2, and IFITM3, as well as IFITM5 and IFITM10 with an unknown role in immunity (75). Brass et al. first described IFITM proteins as antiviral factors that target the early life cycle steps of several viruses (76). HCV infection could be restricted by IFITM proteins by targeting the endocytosed HCV virion for lysosomal degradation (77). However, our previous study indicated IFITM2 was shuttled by exosomes to DCs and inhibit IFN-α synthesis *via* inhibiting phosphorylation of extracellular signal-regulated kinase (ERK), TANK-binding kinase 1 (TBK1), and interferon regulatory factor 3 (IRF3), thereby blocking anti-HBV efficacy of exogenous IFN-α in chronic HBV infection (40). Thus, down-regulating IFITM2 may be a potential strategy to improve the therapeutic response to IFN-α treatment.

Most negative regulators function in the various biological processes in IFN production and signaling pathways, including inhibit IFN expression, disturb IFNAR function, and suppress the expression of ISGs which serve as anti-HBV molecules (Table 1).

### USP18 and ISG15

Ubiquitin-specific protease 18 (USP18, also known as UBP43) and Interferon stimulated gene 15 (ISG15) have been studied extensively for their negative regulation of type I IFN signaling. Various studies showed that ISG15/USP18 signaling activation is involved in IFN therapy non-response in both HBV and HCV patients (78, 79). Responding to viral infection or IFN stimulation, both USP18 and ISG15 are strongly upregulated (80). USP18 belongs to the ubiquitin protease family, which is responsible for removing ubiquitin or ubiquitin-like proteins from their conjugated substrates (81). ISG15 is a ubiquitin-like modifier that binds other proteins in a process called ISGylation, and its antiviral activity was first observed using a recombinant chimeric Sindbis virus and IFNAR^−/−^ mice model (82). ISG15 conjugated to various cellular substrates *via* an enzymatic cascade: E1 activating enzyme UbE1L (83), E2-conjugating enzyme UbCH8 (84, 85), and various E3 ligase (86, 87). ISG15 targets host and viral proteins and impacts diverse cellular pathways, including RNA splicing, chromatin remodeling/polymerase II transcription, cytoskeleton organization and regulation, stress responses, and translation mainly in a conjugation-dependent manner (88, 89). By deISGylating ISG15, USP18 was stabilized by unconjugated ISG15 *via* preventing its S-phase kinase-associated protein (SKP2)-induced ubiquitylation, therefore mediates downregulation of type I IFN signaling during HBV infection (41). Independent of its deconjugating activity, USP18 also competes with JAK1 for binding to the IFNAR2 and therefore impede IFN signaling (42). Because USP18 and ISG15 are inducible by viral factors, these inhibitory proteins may be important mediators in reducing IFN therapy efficacy by negatively regulating IFN-α signaling and host immune against chronic HBV infection.

### SOCSs

In addition to USP18 and ISG15, the suppressor of cytokine signaling (SOCS) proteins and cytokine-inducible SH2 (CIS) also play important roles in downregulating IFN-α signaling, particularly the JAK-STAT signaling pathway (90), and suppress type I IFN production. To date, SOCS/CIS family includes eight proteins (SOCS 1-7 and CIS) which sharing similar structures: a central Src-homology 2 (SH2) domain, a C-terminal SOCS box, and a highly variable N-terminal region (91). The SOCS box functions to recruit an E3 ubiquitin ligase complex, so the SOCS box-containing proteins can act as substrate-recognition modules to mediate the polyubiquitination and subsequent degradation of substrate proteins by the 26S proteasome (92). Responding to IFN signaling and cytokine stimulation, SOCS 1-3 and CIS proteins are induced and negatively regulate the JAK-STAT-mediated cytokine signal responsible for its production in a classic negative feedback loop (30, 45), while SOCS4-7 primarily focus on regulating the growth factor receptor signal (93, 94). SOCS1 and SOCS3, the most widely studied proteins in this family, possess a kinase inhibitory region (KIR) at the N-terminal region that inhibits JAK tyrosine kinase activity by acting as a pseudo-substrate (95). At the same time, SOCS1 is related to IFNAR1 specific signals, thereby abrogating tyrosine phosphorylation of STAT1 and reducing the duration of antiviral genes expression (46). Apart from regulating the IFN-α signaling pathway, SOCS proteins can suppress type I IFN production by binding and degrading key molecules in the TLR signaling pathway, including myeloid differentiation factor 88 (MYD88)-adaptor-like (Mal) protein (43) and IRF7 (44). HBV increased SOCS1 and SOCS3 expression and resulting in sustained STAT3 activation (96, 97). Thus, the SOCS family, especially SOCS1 and SOCS3, inhibit both type I IFN production and IFN-α signaling pathway in HBV infection and provides targets for improving IFN-α-mediated antiviral effect.

### EGFR

Epidermal growth factor (EGF) receptor (EGFR) is a transmembrane protein that belongs to the ErbB family of receptors and is well-studied for its association with a number of cancers. EGFR has recently been shown to be a host entry cofactor triggering HBV internalization (98). Inhibition of EGFR activity enhances the antiviral efficacy of IFN-α by both inducing SOCS3 expression to reduce IFN-α-induced STAT3 activity and enhancing STAT1-mediated antiviral response. Moreover, EGFR inhibitors inhibited the replication, antigen syntheses, and cccDNA reservoir of HBV (47). Thus, EGFR inhibitors could be an important candidate that facilitates IFN-α therapy against HBV infection.

### PTPs

Protein tyrosine phosphatases, known as PTPs, is a large family that encodes enzymes that are divided into the classical, phosphotyrosine (pTyr)-specific phosphatases and the dual specificity phosphatases (DSPs) (99), regulating the phosphorylation state of many important signaling molecules. The overexpression of HBx or NF-κB led to increased SHP2 expression, which is a ubiquitously expressed PTP, *via* NF-κB binding to the *Shp2* promoter (100). SHP2, encoded by Ptpn11, containing two SH2 domains and acts as a negative regulator of IFN-induced STAT activation in a protein kinase Cβ (PKCβ)-dependent pathway (48, 49). PTP1B is another PTP protein and is capable of binding and dephosphorylating IFNAR1 and thereby suppress type I IFN signaling in human cells. PTP1B inhibitors robustly augmented the type I IFN-mediated antiviral effects against HCV (50). However, the knowledge on PTP1B expression and its function needs further study in chronic HBV infection. Currently, it is estimated that as many as 125 PTP genes are in the human genome, and most of them have not been identified (101). Thus, additional studies are required to identify PTP proteins that suppress the IFN-α signal pathway and therefore attenuate therapy in chronic HBV infection.

### Other Molecules

Apart from the above-mentioned molecules, other molecules involved in the down-regulation of IFN therapy efficacy include NEIL3, TDG, and NT5C3, but the exact biological mechanisms underlying the influence of these molecules in IFN-α treatment response remain to be determined. It is reported that positive response to IFN-α treatment in CHB patients is associated with a lower level of NEIL3 and thymine DNA glycosylase (TDG), two intra-hepatic base excision repair (BER) genes (102), but the underlying mechanism is unknown. MiR-122, a miRNA that has been demonstrated to inhibit HBV replication by directly targeting the HBV pre-genomic RNA sequence or by indirectly modulating HO-1 and CCNG1/p53 pathways (103), could be sequestered by an ISG, NT5C3 (51). Therefore, NT5C3 could efficiently inhibit miR-122 and suppress IFN therapeutic efficacy. However, the function of NT5C3 and miR-122 need further confirmation in clinical research. In addition, numerous molecules have been shown to negatively regulate type I IFN signaling, including DCST1, PIAS1, and so on (32, 104), although further studies are needed to clarify their function in HBV infection.

## Role of Cytokines, Chemokines, and miRNAs in Negative Regulation of IFN-α Therapy in CHB Infection

### Peripheral IFNAR

In general, the anti-HBV activity of type I IFN is mediated through binding to their unique receptors, following by JAK-STAT pathway activation and ultimately ISGs production. Cell-surface IFNAR consists of 2 major subunits: IFNAR1 and IFNAR2. However, soluble IFNAR was reported as an inhibitory factor of IFN therapy in both HBV and HCV infection (52, 105). The expression of IFNAR1 and IFNAR2 in lymphocytes and monocytes was increased in CHB patients which was negatively correlated to plasma glutathione S-transferase (GST), a class of cytosolic enzyme participated in the protection of cell from reactive oxygen species (ROS), suggesting that oxidative stress play an important role in the upregulation of IFNAR in CHB patients (37, 52). Oxidative stress could modulate protein misfolding and then disrupt the biological protein conformation. Therefore, oxidative stress may influence IFNAR function, further studies are required to find out the mechanism.

### TNF-α

TNF is a pleiotropic cytokine that exerts homeostatic and pathogenic bioactivities (106). Elevated levels of TNF-α are observed in serum and hepatocytes of patients with acute or chronic hepatitis B (107), possibly through ERKs and NF-κB pathway (108). TNF-α may play a role in downregulating HBV replication in hepatocytes by non-cytopathic mechanisms and synergizing with IFN in suppressing viral replication (109, 110). On the other hand, TNF-α was reported as a negative regulator during IFN-α production determined by HBsAg (55). It is also reported that the injections of synthetic TNF-α could inhibit IFN-α-induced signals in the liver and both SOCS3 and SHP2 contributes to the inhibitory effect (53). However, anti-TNF-α-agents may lead to enhanced HBV replication and cause reactivation of HBV infection in HBsAg carrier and Occult carrier (anti-HBc+) patients (111). Consequently, the ultimate impact of TNFα-mediated effects on IFN therapy against HBV is a question that remains to be resolved.

### IL-10

Interleukin 10 (IL-10), also known as cytokine synthesis inhibitory factor (CSIF), is a pleiotropic cytokine with anti-inflammatory and immunosuppressive activities. HBcAg stimulated IL-10 production by CD4+ and CD8+ cells, as well as by monocytes from CHB patients, which serves as a viral strategy to downregulate the host immune response and allow viral persistence in the host (112). HBsAg inhibited the production of IFN-α by pDCs through the induction of monocytes that secreted IL-10 (55). IL-10 also suppresses NK cell IFN-γ production without altering cytotoxicity or death-ligand expression (56). In addition, SOCS1 and SOCS3 are both produced in response to IL-10, and both function as negative regulators during JAK-STAT signaling (54). On the other hand, evidence suggests that heterogeneity in the promoter region of the IL-10 gene has a role in determining the initial response of HBV infection to IFN-α therapy. IL-10 variants were more frequent among virologically sustained response (SR) compared with non-responders (NR) to IFN-α-2b. Carriage of the−592A allele,−592A/A genotype, and−1082/1819/-592 ATA haplotype was associated with SR (113).

### IL-8

Interleukin 8 (IL-8), or chemokine (C-X-C motif) ligand 8 (CXCL8) is a pro-inflammatory chemokine produced by various cell types to recruit leukocytes to the sites of infection or tissue injury (114). It has been demonstrated that serum IL-8 amounts is elevated during HBV reactivation, and HBeAg-negative patients had significantly higher levels of IL-8 transcripts in the liver than HBeAg-positive patients (115). Accumulating evidence indicates that IL-8 may contribute to counteracting IFN-α antiviral action (116, 117). During the IFN challenge, IL-8 expression induced by HBV can impair the ability of endogenous IFN-α to inhibit early stages of viral replication, thus facilitate viral persistence, and can also contribute to the poor response to IFN-α treatment (57). IL-8 is induced by HBV through various mechanisms, but the mechanism underlying the IL-8 affecting the anti-HBV function of IFN-α therapy still needs further discussion.

### IL-28B

Interleukin*-*28B (IL-28B), also known as IFN-λ3, has been reported by numerous studies that its genetic polymorphisms are related to the therapeutic efficacy of PEG-IFN-α. Sonneveld et al. reported the proportions of IL-28B genotypes were 77, 19, and 5% for AA/AG/GG at rs12980275 and also for CC/CT/TT at rs12979860, respectively (118). rs12980275 genotype AA and rs12979860 genotype CC were associated with a higher probability of HBeAg seroconversion (118). A meta-analysis summarized that IL-28B rs12979860 CC genotype and rs8099917 TT genotype indicated a better treatment response than non-CC and non-TT genotypes for PEG-IFN-α in patients with CHB (58).

### miRNAs

MicroRNAs (miRNAs) are a class of non-coding small RNAs that play important roles in modulating gene expression mainly by base-pairing to the 3' translational region (3' UTR) of mRNAs. miRNAs have been reported to be widely involved in antiviral immunity and some studies show miRNA plays a role in HBV-host interaction. HBV could change the expression profiles of cellular miRNA and escape host immune clearance (119). It was shown that nearly 90% of the total miRNAs in the liver are comprised of ~10 miRNAs and miR-122 is the major contribution with 50–70% of the miRNA profile (120). Zhang et al. identified 11 response-related miRNAs, including let-7a, miR-30a, miR-1290, miR-106b, miR-1224-5p, miR-939, miR-1281, miR-198, let-7f, miR-22, and miR-638, by performing miRNA microarray analysis of plasma samples and liver biopsy samples from CHB patients who received IFN therapy and using the OneR feature ranking and incremental feather selection method to ultimately establish a prediction model for predicting the initial response of HBV patients receiving IFN injections (121). Not all of the 11 miRNAs in the predicted model is negatively associated with the IFN-α therapy efficacy. Results obtained by Tan et al. suggest that the aberrant expression of miRNAs was associated with different therapy outcomes, and monitoring the fluctuation patterns of miRNAs was important for predicting the IFN-α therapeutic effects. They also identified 11 miRNAs to be associated with the effect of HBV therapy, namely, let-7d-5p, let-7f-5p, let-7i-5p, miR-122-5p, miR-126-3p, miR-1307-3p, miR-181a-5p, miR-21-5p, miR-425-5p, miR-652-3p, and miR-320a-3p, with 9 miRNAs significantly declined after therapy in the responder group: let-7d-5p, let-7f-5p, let-7i-5p, miR-126-3p, miR-1307-3p, miR-181a-5p, miR-21-5p, miR-425-5p, and miR-652-3p (122).

miRNAs could modulate the IFN-α therapy effect in multiple processes, including type I IFN production, IFN signaling pathway, as well as ISGs transcription and translation. The effect of miRNA on IFN and its signaling pathway has been well-illustrated in many reviews (123, 124), indicating that miR-130a, let-7b, miR-26a, miR-24, miR-146a, and many other miRNAs could down-regulate the effect of IFN therapy. However, only a few miRNAs are studied extensively in CHB patients. HBV infection promotes miR-146a transcription, which suppressed STAT1 and ultimately attenuated the production of type I IFN-induced antiviral factors, as well as impairing T cell function, resulting in IFN resistance and immune defects during chronic HBV infection (59, 60). miR-3613-3p was upregulated in the CHB patients suppressed IFN-induced anti-HBV immune response by targeting cytidine monophosphate kinase 1 (CMPK1) (61), a kinase responsible for the metabolism of CMP, UMP, and deoxycytidine analogs (125), and ultimately decrease the relative expressions of IFN-α and IFN-β. miR-21 has been reported to be associated with the effect of HBV therapy (122) and promote an anti-inflammatory response by increasing IL-10 production through down-regulating programmed cell death 4 (PDCD4) (126). But the detailed mechanism of miR-21 affecting the anti-HBV effect of IFN-α therapy still needs further investigation.

Some miRNAs exhibit an immune-modulating or IFN therapy enhancing effect in HBV infection. HBeAg could elevate miR-212-3p expression in macrophages, and thereby decrease the production of cytokines through targeting MAPK1 (127). Higher pretreatment plasma miR-301a-3p and miR-145-5p levels were observed in CHB patients who achieved combined response or HBsAg loss following peg-IFN/NA therapy compared to non-responders (120). miR-155 enhances innate antiviral immunity against HBV through promoting JAK/STAT signaling pathway by targeting SOCS1 and mildly inhibits HBV infection in human hepatoma cells (128). In conclusion, miRNAs play an important role in regulating the efficiency of IFN therapy in CHB patients, and could act as predictive factors of IFN therapy outcome, or be used for discovery for better treatment strategies. But the mechanism underlying these miRNAs calls for more attention.

### LncRNAs and circRNAs

Long non-coding RNAs (lncRNAs) are a subset of non-coding RNAs >200 nucleotides in length and have recently been suggested to act as both positive and negative regulators in viral infections utilizing various mechanisms, mediated by their specific sequences or structural motifs that bind DNA, RNA, or protein (129). Recent study suggests that HBx-LINE1, a hybrid RNA transcript of the human LINE1 and the HBV-encoded X gene can serve as a sponge for sequestering miR-122 and may therefore promote HBV expression and replication, but whether HBx-LINE1 function during IFN-α treatment is still unknown (130). On the other hand, lncRNA#32 has been reported to be positively associated with type I IFN signaling in HBV infection by regulating APOBEC3A and APOBEC3G expression (131). Given the important and complicated roles of lncRNAs in IFN anti-HBV treatment, more extensive studies are required to develop a more comprehensive profile of lncRNAs in HBV infection and IFN therapy.

Circular RNAs (circRNAs) are closed long non-coding RNAs and can bind miRNA and act as a miRNA sponge to regulate gene expression, they may also play a role in negatively regulating IFN therapy outcomes. Among top ten circRNAs significantly up-regulated in CHB compared to normal controls, circRNA has-circ-0004812 impairs IFN-induced immune response by sponging miR-1287-5p to regulate Follistatin-related protein (FSTL) 1 in chronic hepatitis B (62, 132). Several circRNAs have been demonstrated to function in HBV infection, including hsa_circ_0005389, hsa_circ_0000038, hsa_circ_0000650, hsa_circ_4099, hsa_circ_0000976, hsa_circ_0007750, hsa_circ_0027089, and hsa_circ_100338, but it is still unknown whether they function in IFN-α therapy of HBV infection (133, 134). Therefore, due to presently poor understanding of circRNAs expression, function and regulation, further studies are desired to identify the mechanisms behind different circRNA regulation patterns associated with IFN-α treatment in HBV infection.

## Host Clinical Parameters In Negatively Regulation of IFN-α Therapy

Patients' clinical characteristics are parameters that provide direct information of liver function and have been used for predicting the efficacy of IFN-α therapy. The appearance or upregulation of several parameters is associated with a lower response rate in IFN-α treatment (Table 2). Published data have also demonstrated that HBV serum markers, including HBsAg, HBeAg, and HBV DNA, are baseline predictors of IFN therapy response, which has been summarized in several reviews (153, 154).

**Table 2:** Involvement of host parameters in reflecting the IFN-α therapy efficacy in CHB patients.

**Host parameters**	**Modulation of IFN-α therapy**	**Possible underlying mechanisms**
Age	Older individuals have lower response to IFN-α treatment than younger individuals (135, 136).	Impairments of host immunity and more advanced liver disease in the elderly may be responsible for a poor response in older individuals (137, 138).
Gender	Females are more likely to have a sustained response to IFN-α (10, 139).	Estrogen may enhance the efficacy of IFN-α therapy (140).
ALT*	High baseline level of ALT may indicate sustained treatment response (10, 139, 141).	ALT is associated with liver injury.
Bile acids	Individuals who have elevated hydrophobic bile acid concentration may have lower response to IFN-α therapy (142).	Bile acids impairs IFN-α treatment by inhibiting Jak1- and Tyk2-phosphorylation and ISGs expression (142).
Obesity	High BMI* and hepatic steatosis are related to a decreased sustained viral response rate to PEG-IFN (143–145).	Reduce type I IFN response through upregulating SOCS3 and leptin; cause other immune dysfunction associated with T cells and B cells (146).
Insulin resistance	Insulin resistance was associated with virological response to HBeAg-positive immune-reactive CHB patients' therapy with IFN-α (147).	Insulin resistance state affect IFN-α efficacy mainly through downregulating IFN-γ, TNF-α and multiple cytokines (147, 148).
Alcohol	IFN-α therapy is ineffective in those who have alcohol abuse.	Alcohol decrease T cells activation and function and impairs IFN production (149); inhibit IFN-γ-signaling through JAK-STAT1 pathway (149).
Anti-IFN antibodies	Anti-IFN antibodies negatively influence the antiviral effect at early stages of the IFN-α therapy (150–152).	Anti-IFN antibodies may attenuate IFN-α therapy *via* neutralizing effect (150–152).

* *ALT, alanine transaminase; BMI, body mass index*.

### Age

Patient age is a host factor that is related to IFN-α therapy responsiveness in Chronic HBV infection. Generally, it is believed that younger individuals are more likely to develop a sustained virological response to IFN-α treatment than older individuals. Bonino et al. reported that patients under 40 years of age had higher combined response rates than patients over 40 years of age, regardless of which treatment they received (with or without lamivudine) (*p* < 0.024; OR: 1.3 per 10-year decrease; 95% CI: 1.0–1.7), indicating that aging imparts a negative influence in the IFN-α treatment efficacy (135). Similar results were showed in other clinical researches (136). Aging enhances the vulnerability to liver injury mainly by increasing oxidative stress and inflammatory response, accelerating cellular senescence, as well as suppressing mitochondrial function (137). A retrospective study showed that age parameters were also associated with significant histological abnormalities in patients with persistently normal ALT levels (PNALT) (155). Moreover, impairments of cellular, humoral, and innate immunity in the elderly may be another explanation responsible for decreased virological response to IFN-α therapy in older patients (138).

### Gender and Hormone

Gender is another important host factor associated with IFN-mediated antiviral efficacy against HBV infection. It is summarized that females are more likely to achieve HBeAg seroconversion after IFN therapy comparing to males (10, 139), which may be an effect of sex hormones. Androgen-androgen receptor (AR) complex activates HBV RNA transcription through binding to the 2 response elements inside Enh I, while estrogen and/or its receptor suppressed HBV replication (156). The expression level of estrogen receptors (ESRs) in the cytosol of peripheral blood mononuclear cells (PBMCs) was significantly lower in patients with CHB than in healthy controls, and ESR1 mRNA expression at week 4 of PEG IFN-α treatment was a better indicator for treatment response although the underlying mechanism is still unknown (140). Further studies are required to clarify the effect of sex hormones in IFN-α therapy.

### ALT Levels

Alanine transaminase (ALT) is a transaminase enzyme and is clinically measured as a biomarker of liver injury. ALT is associated with significant histological characteristics to some extent in HBeAg-positive and -negative patients and its normalization are related to favorable long-term CHB infection treatment outcomes. Several studies indicated that a high baseline ALT level can be a predictor of sustained treatment response (10, 139, 141).

### Bile Acids

Bile acids are steroid acids and were found to be related to the IFN-α treatment. Elevated hydrophobic bile acid concentration impairs IFN-α treatment in patients with CHB by inhibiting Jak1- and Tyk2-phosphorylation, resulting in a decreased mRNA and protein expression of ISGs, including myxovirus resistance protein A (MxA) and dsRNA-activated protein kinase (PKR) (142). The uptake of bile acid into hepatocytes is induced by Na^+^-taurocholate cotransporting polypeptide (NTCP), which also plays an essential role in HBV entry into the target cells. HBV infection may promote the expression of genes involved in lipid and bile acid metabolism by binding to NTCP and limits its function, thus promoting compensatory BA synthesis (157).

### Obesity and Hepatic Steatosis

High body mass index (BMI) or obese status is related to the presence of steatosis in CHB patients. Clinical researches showed that the occurrence of hepatic steatosis is significantly high in CHB patients, decreasing the sustained viral response (SVR) rates to PEG-IFN (143–145). Recent studies indicated that obesity attenuates and prolongs the type I IFN response that shows antiviral inefficacy. Obese individuals have a high level of blood leptin, resulting in the upregulation of the SOCS3. The elevated leptin and SOCS3 levels could reduce type I IFN response and cause other immune dysfunction associated with T cells and B cells in people with obesity (146).

### Insulin Resistance

The relationship between HBV infection and insulin resistance (IR) has not been completely elucidated, but it is reported that HBV infection was associated with increased IR (158). Researches illustrated that IR was associated with virological response to HBeAg-positive immune-reactive CHB patients' therapy with IFN-α mainly through downregulation of IFN-γ, TNF-α and multiple cytokines (147, 148). On the other hand, IFN-α was reported to be a catastrophic feature of the diabetic islet microenvironment, although the conclusion is controversial (159, 160). Therefore, the relationship of diabetes and IFN-α treatment is complicated and need further study.

### Alcohol

Alcohol abuse causes rapid progression of liver disease in HBV infected patients and allows HBV to persist chronically mainly by increasing HBV replication, inducing oxidative stress, and suppressing the immune response. As reported, alcohol reduces the number of T-cells and changes the ratio of T-cell types, resulting in decreased T-cell activation and function, thereby impair IFN production (149). On the other hand, the combination of HBV and ethanol metabolites impairs IFN-γ-signaling through the JAK-STAT1 pathway (149). Moreover, alcohol and HBV synergistically promote hepatic steatosis (161), which has been reported as another negative host factor of IFN therapy against HBV infection.

### Anti-IFN Antibodies

Endogenous anti-IFN antibodies have been reported to be stimulated after the treatment with IFN. Multiple studies indicated that anti-IFN antibodies may negate the antiviral effects of IFN-α by neutralizing the effect (150–152). Anti-IFN was significantly more likely to develop in patients who received lower doses (2.5 or 5 MU/m^2^) of IFN than in those who received a higher dose (10 MU/m^2^) (151). Another study showed that the anti-IFN activity may negatively influence the effect of the IFN therapy on CHB patients at the early stages of the therapy, but the appearance of the anti-IFN activity at the end of a long-term IFN therapy has no significant influence on the treatment outcome (152).

## Concluding Remarks

Improving the efficacy of IFN-α for chronic HBV infection as well as understanding the mechanisms underlying HBV- and host-mediated resistance to IFN-α therapy remains a challenge. Identifying and inhibiting the pathways or molecules that negatively regulate IFN-α signaling and therapy, as well as finding host parameters that predict the response of patients with CHB to antiviral therapy are obvious ways to improve the efficacy of IFN-α. Diversity negative factors function at many levels of IFN signaling: the recognition of HBV by the host immune system, the production of IFNs, the recognition of IFNs by their receptors, and the JAK-STAT signaling pathway.

IFN-α therapy prescript with inhibitors of negative regulators is a potential therapeutic strategy for achieving a clinical cure. However, the concentration, duration, and tissue specificity of the negative regulators are important parameters in their synergistic effect, and we need to know more. We may need further investigation to identify an appropriate level and ratio among the inhibitors of different regulators to suitably inhibit the negative regulators, limit the toxicity, and enable a return to homeostasis in a majority of CHB patients. For negative regulators with different duration of effects, for example, before, during, and after the IFN-α therapy, the inhibition strategy may be appropriate and desirable to be flexible. Inhibitors are better to function at the same biological procedure during the IFN-α therapy, so that enhancing its anti-HBV effect accurately. For negative regulators mainly expressed in the liver tissue, the effect of inhibition should be limited in the liver to minimum side effects.

Negative host factors also serve as predictive factors for predicting the IFN-α therapy treatment outcome of CHB patients before and during the therapy. Host parameters including age, gender, ALT level, and BMI have been recommended in guidelines and widely used in deciding whether to administer IFN-α therapy for CHB patients. miRNA and cytokines are fluctuating factors important for predicting the IFN-α therapeutic effects that worth and easy to be monitored. Some negative factors only saw upregulation in liver cells with the procedure of liver biopsy, thus, these molecules should be put last on the list of predictors.

In addition to the host negative factors mentioned above, host genetic factors can provide unexpected regulators of IFN-α therapy and influence the therapeutic effects in complicated manners, which has been well-summarized in other reviews (162, 163). Single nucleotide polymorphisms (SNPs) could also serve as potential markers that predict hepatitis B patient response (164). When combined with basic clinical parameters and other genetic and epigenetic markers, more reliable treatment indexes can be developed and ultimately applied to the clinic.

Thus, as discussed above, IFN therapy needs more novel and reliable biomarkers to improve management, and novel combination strategies to substantially increase the efficacy of treatment of CHB patients.

## Author Contributions

JW, LD, and HT contributed to the design of the review. JW and LD searched the database and wrote the first draft of the manuscript. All authors contributed to manuscript revision, read, and approved the submitted version.

## Funding

This work was supported by the National Natural Science Foundation (Grant No. 82172254) and 1.3.5 project for disciplines of excellence. West China Hospital, Sichuan University (Grant No. ZYGD20009).

## Conflict of Interest

The authors declare that the research was conducted in the absence of any commercial or financial relationships that could be construed as a potential conflict of interest.

## Publisher's Note

All claims expressed in this article are solely those of the authors and do not necessarily represent those of their affiliated organizations, or those of the publisher, the editors and the reviewers. Any product that may be evaluated in this article, or claim that may be made by its manufacturer, is not guaranteed or endorsed by the publisher.
